# Diet-Sensitive Sources of Reactive Oxygen Species in Liver Mitochondria: Role of Very Long Chain Acyl-CoA Dehydrogenases

**DOI:** 10.1371/journal.pone.0077088

**Published:** 2013-10-07

**Authors:** Ariel R. Cardoso, Pâmela A. H. B. Kakimoto, Alicia J. Kowaltowski

**Affiliations:** Departamento de Bioquímica, Instituto de Química, Universidade de São Paulo, São Paulo, São Paulo, Brazil; Boston University, United States of America

## Abstract

High fat diets and accompanying hepatic steatosis are highly prevalent conditions. Previous work has shown that steatosis is accompanied by enhanced generation of reactive oxygen species (ROS), which may mediate further liver damage. Here we investigated mechanisms leading to enhanced ROS generation following high fat diets (HFD). We found that mitochondria from HFD livers present no differences in maximal respiratory rates and coupling, but generate more ROS specifically when fatty acids are used as substrates. Indeed, many acyl-CoA dehydrogenase isoforms were found to be more highly expressed in HFD livers, although only the very long chain acyl-CoA dehydrogenase (VLCAD) was more functionally active. Studies conducted with permeabilized mitochondria and different chain length acyl-CoA derivatives suggest that VLCAD is also a source of ROS production in mitochondria of HFD animals. This production is stimulated by the lack of NAD^+^. Overall, our studies uncover VLCAD as a novel, diet-sensitive, source of mitochondrial ROS.

## Introduction

A prominent increase in obesity has been observed over the last decades, often associated with liver steatosis, characterized by triacylglicerol accumulation in hepatocytes [1-4]. Triacylglicerol accumulates due to an imbalance between lipid oxidation and synthesis, absorption and secretion [2]. Lipid catabolism in the liver occurs primarily by acyl-CoA oxidases in peroxisomes and acyl-CoA dehydrogenases in mitochondria [4-6]. Both peroxisomes and mitochondria are important intracellular sources of reactive oxygen species (ROS), which have been shown to be involved in non-alcoholic fatty liver diseases and non-alcoholic steatohepatitis [4,7]. Imbalance between ROS production and antioxidant systems may promote DNA damage, lipid peroxidation and cell death [2,4,7], which worsen steatosis and promote the transition to more severe conditions, including cirrhosis and hepatocellular carcinoma [2,4].

The primary ROS generated by mitochondria is the superoxide anion radical (O_2_^.-^), a by-product of respiration [8-10]. Superoxide dismutases then dismutate O_2_^**.** -^ to hydrogen peroxide (H_2_O_2_) [11-13] which is more diffusible and stable, and therefore can be detected more readily and have more distant cellular targets [8,9,14].

Mitochondrial O_2_^**.** -^ formation is usually attributed to electron leakage at respiratory chain complexes I and III [8,10,15]. However, it is important to remember that mitochondria are extremely rich in oxidation-reduction reactions, and thus have many other potential sources of O_2_^**.** -^ and other ROS. In this regard, flavoproteins have increasingly been recognized as oxidant sources in mitochondria [10,14,16–20]. A well-studied example is dihydrolipoamide dehydrogenase (or dihydrolipoyl dehydrogenase), a subunit of pyruvate and α-ketoglutarate dehydrogenases, which can produce significant quantities of mitochondrial O_2_^**.** -^ [17-20]. Glycerol-phosphate dehydrogenase is also a well-established mitochondrial source of oxidants [19]. However, other potential sources of mitochondrial ROS are still more poorly explored, including acyl-CoA dehydrogenases, the electron transferring flavoprotein (ETF) and the electron transferring flavoprotein Q oxidoreductase (ETF-DH) [9,14,21,22], all involved in lipid catabolism, and therefore potentially important in steatosis.

In mitochondria, fatty acid catabolism is regulated mainly by the transport of long chain acyl-CoA groups into the mitochondrial matrix by carnitine palmitoyltransferase (CPT 1), inhibited by extramitochondrial malonyl-CoA [4,23]. Dehydrogenation performed by acyl-CoA dehydrogenases can also be a rate-limiting step [6]. Acyl-CoA dehydrogenases [5] include five enzymes involved in fatty acid metabolism: short chain acyl-CoA dehydrogenase (SCAD), medium chain acyl-CoA dehydrogenase (MCAD), long chain acyl-CoA dehydrogenase (LCAD) and two forms of very long chain acyl-CoA dehydrogenase (VLCAD_1_ and VLCAD_2_) [5,6,24]. VLCADs consist of 73 kDa homodimers, while all other acyl-CoA dehydrogenases are 43 kDa homotetramers with one non-covalent FAD per subunit [5,25-28].

In peroxisomes, fatty acid catabolism is promoted by the acyl-CoA oxidase superfamily, which is believed to display similar catalytic mechanisms to acyl-CoA dehydrogenases [5,6,29]. They differ in the transfer of electrons: while acyl-CoA dehydrogenases transfer electrons to the ETF, acyl-CoA oxidases reduce molecular oxygen to H_2_O_2_ [5,6,30]. H_2_O_2_ is thus a physiological product of fatty acid oxidation by peroxisomes, in particular in liver [31]. It is debatable, however, if the H_2_O_2_ produced can leak from peroxisomes, due to the high quantities of catalase and H_2_O_2_ scavengers present in these organelles [32].

In this study, we evaluated the effects of a high-fat diet on liver ROS generation. Interestingly, our results indicate that mitochondrial acyl-CoA dehydrogenases may potentially be a significant, diet-sensitive, source of ROS.

## Materials and Methods

### Reagents

The following compounds were purchased from Sigma-Aldrich^®^: percoll, sucrose, BSA, HRP, Hepes, palmitoyl-CoA, octanoyl-CoA, butyryl-CoA, malonyl-CoA, palmitoyl-carnitine, NAD^+^, NADH, succinate, ADP, antimycin A, rotenone, oligomycin, CCCP, valinomycin. Safranin O and Amplex Red were purchased from Amresco^®^ and Molecular Probes^®^, respectively. Primary antibodies from rabbit against mouse acyl-CoA dehydrogenases were purchased from LSBio^®^ for SCAD, ProteinTech^®^ for LCAD and Santa Cruz^®^ for MCAD and VLCAD, ETF-A and ETF-B from Sigma^®^. Secondary anti-rabbit antibodies were from Calbiochem^®^.

### Animals and diet

All experiments were performed with 6 week old female Swiss mice acquired from our animal facilities (*Biotério de Produção e Experimentação da Faculdade de Ciências Farmacêuticas e do Instituto de Química da USP*), which follows specifications of the Guide for Care and Use of Laboratory Animals, National Press, USA. Experiments were approved by the institutional animal ethics committee. Control and high fat diet (HFD) mice were fed a Rhoster^®^ standard diet, but the water source of the HFD group was supplemented with soy oil (30% v/v) in emulsion with 9 g/L sodium stearoyl lactilate from Purac^®^ [33,34]. We used soybean oil supplementation as a HFD because it induced obesity within 4 weeks without changing serum triacylglycerol, glucose or total cholesterol contents [34]. Soybean oil contains primarily polyunsaturated fatty acids (PUFA) and does not cause liver inflammation or diabetes when used short-term. Animals were treated for one week with this diet, which leads to ingestion of 55% in weight of fat, versus 4% fat in the control group. At this point, control animals weighted 39.70 g ± 1.59, n = 10, while HFD animals weighed 42.20 g ± 0.89, n = 10. HFD mice had lower food consumption: ~1 g per day versus ~3 g per day in the control group. The consumption of water or emulsion was equal at approximately 7 mL per day. After treatment, animals were sacrificed and samples were prepared immediately.

### Histology

Fresh livers were rinsed in isolation buffer, fixated for 18 hours with Zamboni solution [35], dehydrated with ethanol and embedded in historesin from *Leica^®^*. Serial 5 µm sectioning was obtained using a *Leica^®^* RM 2145 microtome and sections were stained with Hematoxylin and Eosin (H&E) or Hematoxylin with oil red O to stain neutral lipids [36]. Images were acquired with an Axioskop microscope and Axiocam from Carl Zeiss^*®*^.

### Liver extracts and mitochondrial preparation

**Figure 2:**
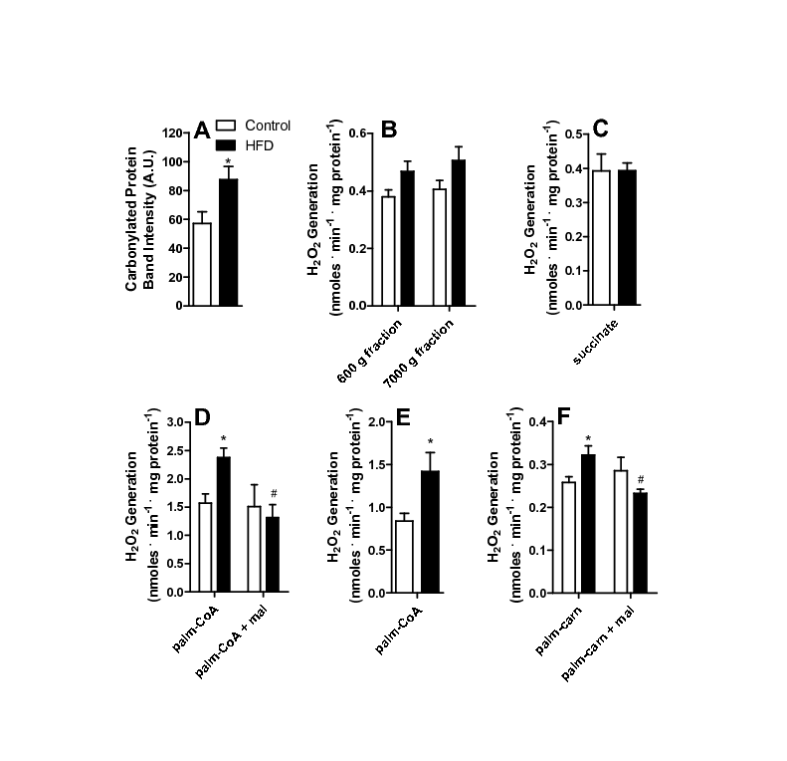
H_2_O_2_ release from liver homogenate fractions. Liver homogenates from control and HFD animals were prepared as described in Materials and Methods and incubated in buffer containing 150 mM KCl, 2 mM MgCl_2_, 1 mM EGTA, 1 mM KH_2_PO_4_, 10 mM Hepes, 0.1% BSA, pH 7.4. 5 μM Amplex Red and 1 U/mL HRP were present to measure H_2_O_2_. (A) Carbonylated proteins were detected as described in Materials and Methods; (B) H_2_O_2_ release from the total (600 g) and mitochondrially-removed (7000 g) fractions; (C) Mitochondrial fraction incubated in the presence of 2 mM succinate; (D) Mitochondrial fraction incubated in the presence of 50 μM palmitoyl-CoA (palm-CoA) and 50 μM malonyl-CoA (mal), where indicated; (E) Percoll-purified mitochondrial fractions incubated in the presence of 50 μM palmitoyl-CoA; (F) Mitochondrial fraction incubated in the presence of 50 μM palmitoyl-carnitine (palm-carn) and 50 μM malonyl-CoA (mal); *, p < 0.05 versus control, ^#^, p < 0.05 versus HFD in the absence of malolyl-CoA, n = 6 per group.

Immediately after sacrifice, livers were placed in cold (4°C) isolation buffer containing 300 mM sucrose, 2 mM EGTA and 10 mM Hepes, pH 7.2. The tissue was minced with surgical scissors and then extracts were obtained using a mechanical potter. Subsequently, the extract was centrifuged at 4°C for 5 minutes at 600 g; nuclei were pelleted in this step and the supernatant was collected (600 g fraction). The 600 g homogenate was then centrifuged at 4°C for 5 min at 7000 g; mitochondria and soft particles are sedimented at this step and the supernatant was collected (7000 g fraction). Mitochondria were resuspended in buffer and kept over ice. To separate mitochondrial preparations from peroxisomes in Figure 2E, we used a percoll gradient purification [37]. We prepared isolation buffers with 62%, 52%, 19% and 7% percoll, adjusting the pH and concentration of EGTA, sucrose and Hepes. The buffers were gently pipetted in decreasing order and mitochondria were added over the top. Tubes were centrifuged at 4°C for 5 minutes at 7000 g with a fixed angle rotor. Peroxisomes, present above fractions of 19% percoll concentrations, were discarded. Mitochondria were collected at the interface between 52% and 19% percoll with a glass Pasteur pipette, resuspended in isolation buffer and centrifuged at 4°C for 5 minutes at 7000 g to remove excess percoll. Mitochondria were then kept over ice.

### Protein quantification

Experiments were normalized by protein concentration using the Bradford reagent protein assay from Bio-Rad. Calibration curves were prepared with bovine serum albumin and the absorbance was measured with a SpectraMax M3 96 well micro plate reader from Molecular Devices^®^.

### H_2_O_2_ generation

To determine the kinetics of H_2_O_2_ generation, we used a F-4500 Hitachi^®^ fluorimeter with magnetic stirring at ex = 563 and em = 586, 37°C. Samples were added to cuvettes containing experimental buffer: 150 mM KCl, 2 mM MgCl_2_, 1 mM EGTA, 1 mM KH_2_PO_4_, 10 mM Hepes and 0.1% BSA, pH 7.4, containing 5 μM Amplex Red and 1 U/mL horseradish peroxidase (HRP) [9,34]. The oxidation of Amplex Red by H_2_O_2_ (1:1) produces resorufin, which is fluorescent. Calibration curves were conducted with known concentrations of H_2_O_2_. Mitochondria were used at 0.5 mg protein/mL buffer. Amplex Red oxidation *in vitro* without mitochondrial samples was not affected by the presence of palmitoyl-CoA (results not shown).

### Oxygen consumption

O_2_ consumption was measured using an OROBOROS Oxygraph-2K electrode at 37°C, with magnetic stirring. Mitochondria were incubated at 0.5 mg protein/mL in buffer containing 120 mM sucrose, 65 mM KCl, 2 mM MgCl_2_, 1 mM KH_2_PO_4_, 1 mM EGTA, 10 mM Hepes and 0.1% BSA, pH 7.4. To calculate respiratory control ratios (RCR), respiratory rates in the presence of 1 mM ADP were divided by rates in the presence of 1 µg/mL oligomycin, using 2 mM succinate as a substrate. Where used, CCCP concentrations were 1 µM. To determine the ADP/O ratios [38], 100 µM ADP was added. Where used, palmitoyl-carnitine and malonyl-CoA concentrations were 50 µM.

### Mitochondrial inner membrane potential (ΔΨ) measurements

ΔΨ was measured using the fluorescent dye safranin O in a F-4500 Hitachi^®^ fluorimeter at ex = 495 and em = 587, 37°C, with magnetic stirring. Mitochondria (0.5 mg/mL) were added to cuvettes containing 120 mM sucrose, 65 mM KCl, 2 mM MgCl_2_, 1 mM EGTA, 1 mM KH_2_PO_4_, 10 mM Hepes, 2 mM succinate and 0.1% BSA, pH 7.4. After an initial reading, 1 mM ADP was added, followed by 1 µg/mL oligomycin and 1 µM CCCP. Calibration curves were performed in media devoid of potassium salts in the presence of 2 μM valinomycin, by adding known KCl concentrations [9,39].

### Western blotting

Acyl-CoA dehydrogenases were quantified in mitochondrial samples diluted in sample buffer with 4% (v/v) glycerol, 10 mM dithiothreitol, 2% (v/v) SDS, 0.05% bromophenol blue and 40 mM Tris-HCl, pH 6.8. To each well of an SDS-PAGE gel electrophoresis, 30 μg of protein were added. Proteins were transferred to PVDF membranes. Primary antibodies were used at 1:1000 in TTBS buffer with 1% BSA for 1 hour at room temperature and secondary antibodies were used at 1:10000 dilutions in TTBS buffer for 1 hour at room temperature. Images were analyzed using ImageJ software and the area of the bands was calculated. To normalize results, we divided the band area by the total column signal of the membranes died with Ponceau (Bio-Rad).

### Carbonylated proteins

To measure carbonylated proteins we incubated mitochondrial samples with 24% SDS and 40 µM 2,4-dinitrophenylhydrazine for 30 minutes at room temperature avoiding light exposure. The reaction was blocked by vortexing samples with buffer containing 2 M Tris, 30% glycerol and 19% β-mercaptoethanol. Samples was boiled and prepared for western blotting using 1:1000 anti-dinitrophenylhydrazine [40].

### NAD(P)H measurements

NAD(P)H and NAD(P)^+^ were measured using a F-4500 Hitachi^®^ fluorimeter at ex = 332 and em = 464, 37°C, with magnetic stirring. Fresh mitochondria (0.5 mg/mL) were added to buffer containing 150 mM KCl, 2 mM MgCl_2_, 1 mM EGTA, 1 mM KH_2_PO_4_, 10 mM Hepes and 0,1% BSA, pH 7.4. Total reduced NAD(P)H was measured adding 1 µM rotenone, and total oxidized fluorescence levels were measured in the presence of 1 μM CCCP. To calculate the capacity of permeabilized mitochondria to reduce NAD^+^, we used frozen mitochondrial samples incubated in buffer containing 150 mM KCl, 2 mM EGTA, 1 mM KH_2_PO_4_, 10 mM Hepes and 0,1% BSA, pH 7.4 with additions of 0.5 µg/mL alamethicin, 0.5 ng/mL antimycin A, 1 µM rotenone and 50 µM NAD^+^. Fifty micromolar palmitoyl-CoA, octanoyl-CoA or butyryl-CoA were also present, as indicated. A calibration curve was constructed with known concentrations of commercial NADH.

### Statistics

**Figure 3:**
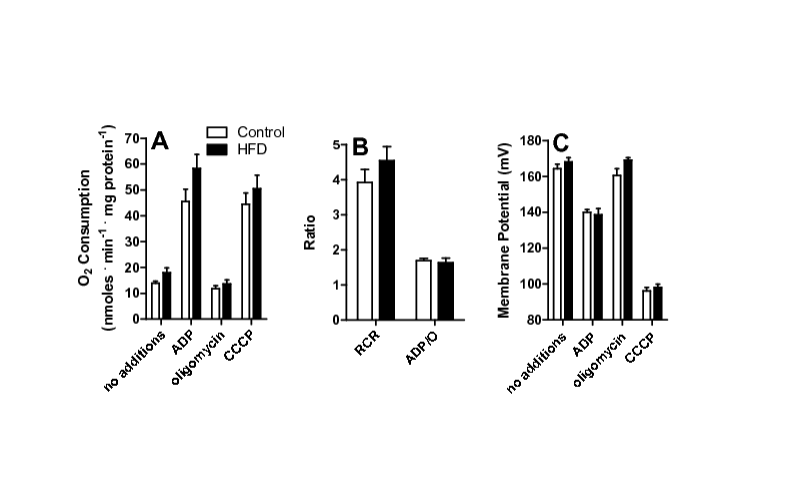
Mitochondrial respiration and oxidative phosphorylation are unchanged by HFD. (A) Mitochondria (0.5 mg protein. mL^-1^) were incubated in 120 mM sucrose, 65 mM KCl, 2 mM MgCl_2_, 1 mM KH_2_PO_4_, 1 mM EGTA, 10 mM Hepes, 0.1% BSA, pH 7.4. Succinate (2 mM), 1 μg/mL oligomycin, 1 mM ADP and 1 μM CCCP were added. Oxygen consumption was measured as described in Materials and Methods. (B) Respiratory control (RCR) and ADP/O ratios were calculated under the experimental conditions of Panel A. (C) Mitochondrial inner membrane potentials were measured under the conditions of Panel A, as described in Materials and Methods, n = 6 per group.

**Figure 4:**
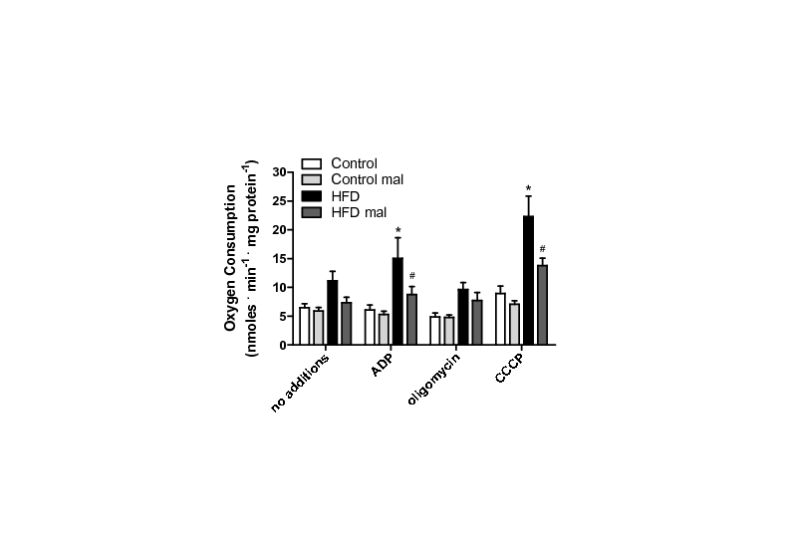
Mitochondrial respiration supported by palmitoyl-carnitine is enhanced in HFD. Mitochondria were incubated under the conditions described for Figure 3, substituting succinate for 50 μM palmitoyl-carnitine. Malonyl-CoA (mal, 50 μM) was present where indicated. *, p < 0.05 versus control, ^#^, p < 0.05 versus HFD in the absence of malonyl-CoA, n = 6 per group.

**Figure 5:**
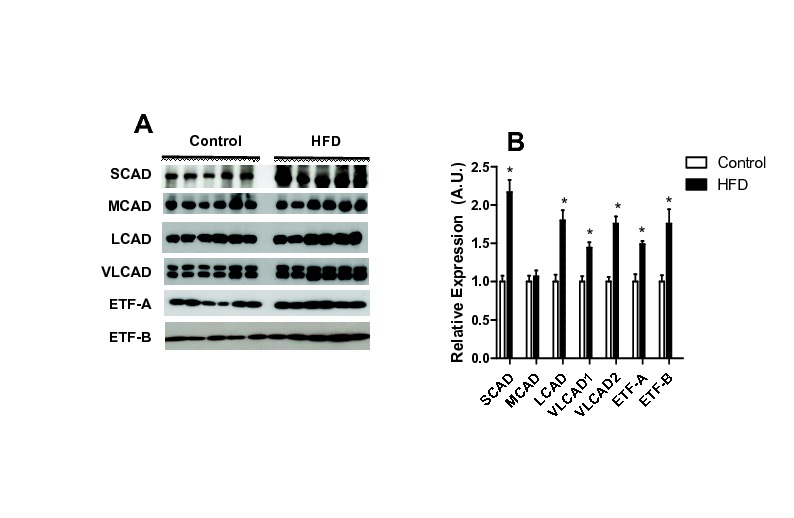
HFD increases the expression of acyl-CoA dehydrogenases isoforms and electron transfer flavoprotein (ETF). Mitochondrial acyl-CoA dehydrogenases and ETF subunits A and B (A-B), were quantified by western blotting as described in Materials and Methods. (A) Representative blots (note that VLCAD has two isoforms), (B) averages ± SEM of densitometries. Where indicated *, p < 0.05 versus control, n = 6 per group.

**Figure 6:**
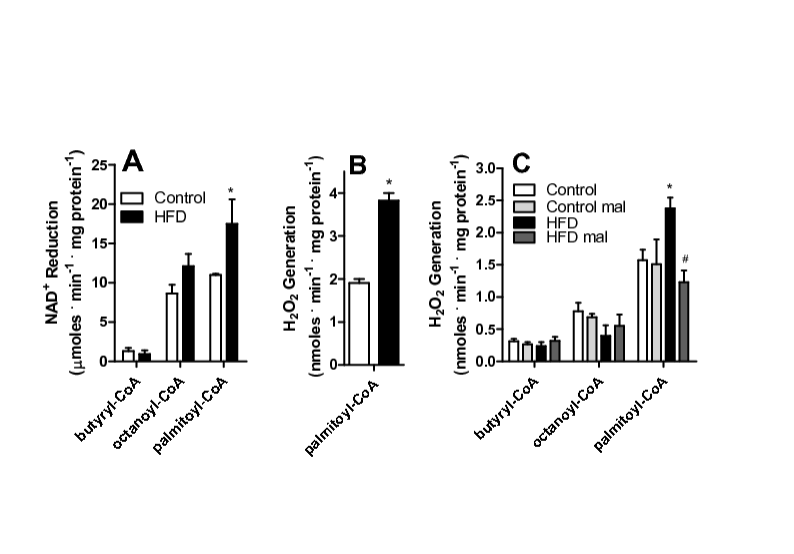
HFD increases ROS release from VLCAD. (A) Frozen and defrosted mitochondria (0.5 mg protein. mL^-1^) were incubated in 150 mM KCl, 2 mM MgCl_2_, 1 mM EGTA, 1 mM KH_2_PO_4_, 10 mM Hepes, 0.1% BSA, pH 7.4. Alamethicin (0.5 μg/mL), 0.5 ng/mL antimycin A, 1 µM rotenone and 50 µM NAD^+^ were added to the incubation media, and NADH fluorescence was monitored as described in Materials and Methods in the presence of 50 μM butyryl-CoA, octanoyl-CoA or palmitoyl-CoA, where indicated. (B) Frozen and defrosted mitochondria were incubated in the same media as Panel A, in the presence of 0.5 μg/mL alamethicin, 5 μM Amplex Red and 1 U/mL HRP. H_2_O_2_ release was measured as described in Materials and Methods. (C) Fresh mitochondria were incubated under the conditions of Figure 3, substituting succinate for 50 μM butyryl-CoA, octanoyl-CoA or palmitoyl-CoA, as shown. Where indicated, 50 µM malonyl-CoA (mal) was present. *, p < 0.05 versus control, ^#^, p < 0.05 versus HFD in absence of malonyl-CoA, n = 6 per group.

**Figure 7:**
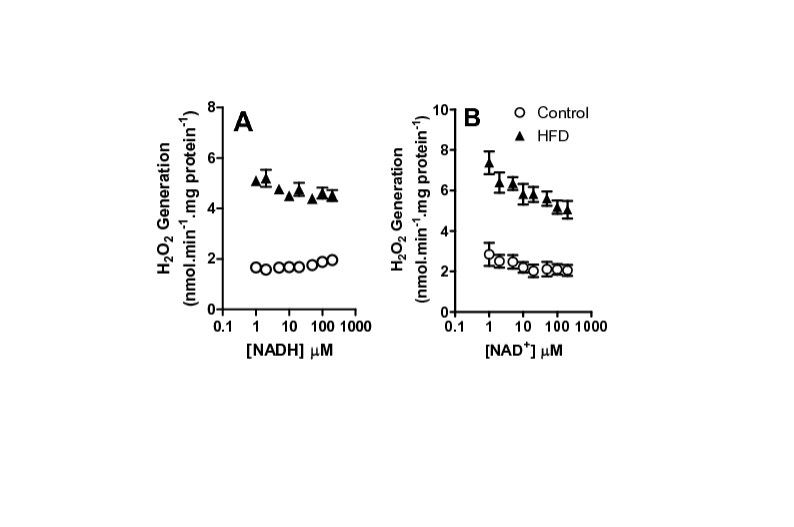
NAD^+^ prevents H_2_O_2_ release by VLCAD. Frozen and defrosted mitochondria (0.2 mg protein. mL^-1^) were incubated under the conditions of Figure 6B, in the presence of varying NADH (Panel A) or NAD^+^ (Panel B) concentrations, as indicated. H_2_O_2_ release was monitored as described in Materials and Methods, n = 6 per group.

A pilot was created to assure that the distributions of measurement variables were normal using the Kolmogorov-Smirnov test. Variances between groups were tested using Bartlett’s test. Both conditions are a prerequisite for the mean tests chosen: two tailed student “t” test (Figure 2, 3; 5B, 6B and 7), one-way ANOVA (Figure 4) and two-way ANOVA (Figure 6A and 6C). When using ANOVA, Bonferroni was used as a post test. All experiments included at least 6 biological repetitions. Statistical significance was considered for p < 0.05.

## Results

**Figure 1:**
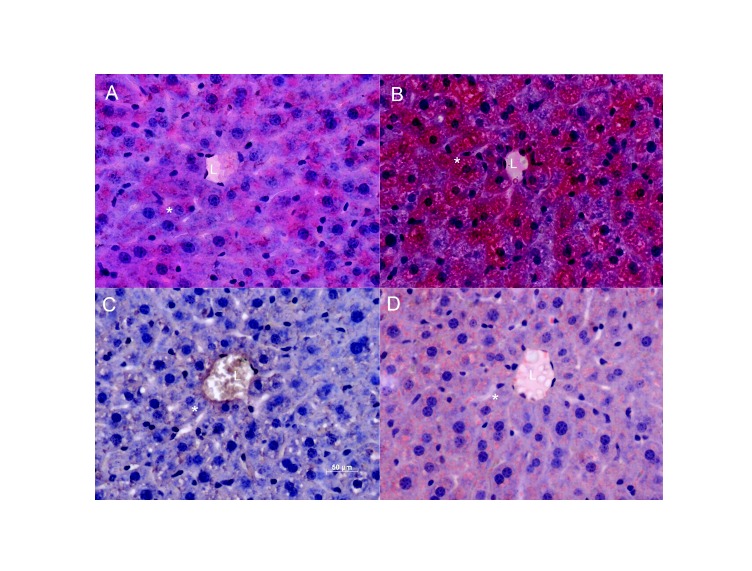
HFD leads to enhanced micro vesicular lipid droplets in livers. Panels A and B: Sections were stained with Hematoxylin and Eosin (H and E). Panels C and D: Oil red O staining shows and HFD livers have more areas marked with this dye in regions near the lumen of vessels. “L” indicates the lumen, and a sample hepatocyte is marked with an asterisk. Images are representative of serial sections from 6 livers per group.

The high fat, short-term, dietary intervention we adopted resulted in increased micro vesicular lipid droplet accumulation in hepatocytes observed by H&E staining (compare Figure 1A, controls, with 1B, HFD). In HFD livers (Figure 1D), more areas stained for neutral lipids using the oil red O dye, mainly in regions near the endothelial lumen, were visible compared to controls (Figure 1C). These results indicate that our HFD animals display significant increases in small lipid droplet content. In addition, no indication of Kupffer cell infiltration in HFD livers was observed, eliminating a role for severe inflammatory processes.

The lipid content changes were accompanied by redox imbalance, as indicated by a significant increase in tissue protein carbonyls (Figure 2A). In order to pinpoint the source of the oxidants responsible for these oxidative modifications, we measured H_2_O_2_ release from liver homogenates of control or HFD animals (Figure 2B). We did not find a statistically significant difference in H_2_O_2_ detection neither in the crude homogenate removed of cell debris and nuclei (600 g fraction) nor in the 7000 g supernatant fraction, which is devoid of mitochondria but contains most other cytosolic components, including peroxisomes. Although cellular homogenates may disrupt the characteristics of ROS release in intact cells and obliterate significant differences between compartments, our results suggest that non-mitochondrial sources of ROS are not significantly altered by HFD, so we focused on analyzing the redox state in isolated mitochondria.

Mitochondrial H_2_O_2_ release was measured from isolated organelles (the pellet obtained by centrifuging at 7000 g) in the presence of respiratory substrates. Interestingly, H_2_O_2_ release in the presence of the respiratory substrate succinate (Figure 2C) was equal in mitochondria from control or HFD animals. Similarly, no differences in H_2_O_2_ release between control and HFD mitochondria were observed with NADH-linked substrates (α-ketoglutarate Control: 0.24 ± 0.01 nmoles . min^-1^ . mg^-1^, HFD: 0.26 ± 0.01; malate/glutamate Control 0.21 ± 0.01, HFD: 0.25 ± 0.04). However, when the activated fatty acid palmitoyl-CoA was present (Figure 2D), H_2_O_2_ generation was significantly more prominent in the HFD mitochondria [9,41]. This difference persisted when L-carnitine was added (results not shown). We noted that L-carnitine interferes with resorufin fluorescence calibration curves and, therefore, results using added carnitine were not quantitatively reliable and avoided throughout this work.

Higher H_2_O_2_ release from liver mitochondrial fractions in response to fatty acids has been attributed to peroxisome contamination in the past [42]. In order to verify if this was the case in our system, in which peroxisome quantities are expected to increase due to the HFD, we performed various tests. First, we repeated the measurements in percoll-purified mitochondria. Although total H_2_O_2_ decreased, the difference between control and HFD persisted (Figure 2E). Second, H_2_O_2_ release was measured in the presence of palmitoyl-carnitine, a mitochondrially-specific substrate. Again, a higher release was observed in HFD samples (Figure 2F). Finally, palmitate-supported H_2_O_2_ release in HFD, but not control mitochondria, was inhibited by malonyl-CoA (Figure 2D and F), which inhibits fatty acid transport into mitochondria, but not peroxisomes. Overall, these results indicate that mitochondrial fatty acid-supported ROS release is enhanced by HFD. This conclusion is further supported by the fact that we found no change in the quantity (measured by Western Blot, not shown) or activity (control: 2.14 mU . g^-1^ ± 0.16; HFD: 2.16 ± 0.12, n = 7, p > 0.94) of the peroxisomal marker catalase between control and HFD mitochondrial samples, indicating that HFD samples were not enriched in contaminating peroxisomes.

Changes in ROS production in mitochondria are often found secondarily to altered electron transport, ATP synthesis or coupling [9,10]. Thus, we measured mitochondrial respiratory parameters (Figure 3). Succinate was used as substrate, since NADH-linked substrates α-ketoglutarate, malate and pyruvate provide poorer respiratory rates in liver mitochondria [9], although results were similar (not shown). We found that respiratory rates under maximum (CCCP), phosphorylating (ADP) or non-phosphorylating (oligomycin) conditions (Figure 3A) were unchanged by HFD. Furthermore, HFD did not change respiratory control (RCR) or ADP/O ratios (Figure 3B). Finally, succinate-supported mitochondrial inner membrane potentials (ΔΨ), a major determinant of ROS production [8,9], were equal in control and HFD groups (Figure 3C), under all respiratory states. 

On the other hand, when respiratory rates were measured using palmitoyl-carnitine as a substrate (Figure 4), significant effects of the HFD were observed. As expected, this activated fatty acid supported respiratory rates lower than succinate (compare Figures 4 and 3A), since electron transport through this pathway is typically limited by maximal β-oxidation rates. However, HFD mitochondria displayed a very significant increase in oxygen consumption rates supported by palmitoyl-carnitine, in particular when respiration is stimulated by ADP or the uncoupler CCCP (Figure 4). These increased respiratory rates were prevented by malonyl-CoA. We consistently find that palmitoyl-carnitine-supported respiration is inhibited by malonyl-CoA, an effect which would not be observed if malonyl-CoA were exclusively an inhibitor of the CoA-carnitine substitution activity of carnitine-palmitoyl transferase I, suggesting it may also inhibit the carnitine/acylcarnitine translocase. Respiratory rates supported by palmitoyl-carnitine were not rate-limited by electron transport, since they were significantly lower than those measured using succinate. This result suggests that maximal fatty acid oxidation capacity is enhanced in HFD mitochondria. In order to further investigate this, we measured the expression of fatty-acid dehydrogenases and the electron transfer flavoprotein (ETF) in our samples. We found that acyl-CoA dehydrogenases were increased up to 2.5-fold relative to control (Figure 5A shows representative blots; Figure 5B quantifies the increase relative to control for SCAD, LCAD and the two forms of VLCAD). In addition, ETF subunits A and B (Figure 5B) were also more highly expressed in HFD mitochondria.

Since expression levels do not always reflect activity, we measured fatty acid oxidation using different size acyl-CoA substrates (Figure 6A). In order to measure fatty acid oxidation promoted by the added substrate alone (and not secondary products), isolated mitochondria were permeabilized with the pore-forming compound alamethicin [17], which promotes the free diffusion of enzymatic products out of mitochondria, leading to their dilution and avoiding the accumulation necessary for these to act as substrates for subsequent metabolic steps. This condition also removes the participation of membrane transport systems, including carnitine palmitoyl transferase, in the phenomena observed. Under these conditions, we measured added NAD^+^ reduction as an indicator of acyl-CoA activity, which is structurally and functionally coupled to subsequent hydroxyacyl-CoA oxidation and NAD^+^ reduction. We found (Figure 6A) that palmitoyl-CoA, but not octanoyl-CoA or butyryl-CoA, induced significantly higher NAD^+^ reduction rates in HFD mitochondria. This demonstrates that, although the expression of many acyl-CoA isoforms is increased by HFD, only the activity of VLCAD, which metabolizes palmitoyl-CoA, is measurably enhanced.

VLCAD is a flavoenzyme, and, as such, a potential source of electron leakage leading to mitochondrial ROS generation [16,19]. The production of ROS by mitochondrial flavoenzymes is often determined by changes in the availability of oxidized nucleotides which physiologically receive electrons. We thus measured NAD(P) quantities and reduction state by following the fluorescence of NAD(P)H (not shown) before and after the addition of rotenone (which promotes reduction) and CCCP (which promotes oxidation). Under our conditions, NAD(P)H quantities were equal in mitochondria from control and HFD animals. Furthermore, NAD(P)H was highly reduced in both samples. Thus, changes in NAD^+^/NADH levels cannot justify solely the increased H_2_O_2_ release observed from isolated HFD mitochondria.

On the other hand, the increases in H_2_O_2_ release observed may be due to the enhanced activity of VLCAD. Indeed, we found that H_2_O_2_ release by permeabilized mitochondria (which lose matrix NAD^+^), was significantly larger in HFD samples when pamitoyl-CoA was added (Figure 6B). The finding that H_2_O_2_ release increases are observed with palmitoyl-CoA in permeabilized mitochondria suggests that ROS generation is occurring at the level of VLCAD (with a possible participation of other structurally-linked enzymes). ETFs are flavoproteins, sources of ROS and their expression is enhanced by HFD. However, although we believe these proteins are important sources of ROS under many conditions, in our animals we cannot detect differences between control and HFD mitochondria respiring on acyl-CoAs of different chain lengths, which indicates that the source of differential electron leakage in control versus HFD mitochondria is probably upstream of the ETF. Indeed, experiments with intact mitochondria (Figure 6C) also indicate that enhanced H_2_O_2_ release specific to HFD mitochondria occurs in the presence of palmitoyl-CoA, in a malonyl-CoA-sensitive manner, while shorter chain fatty acids, which also feed electrons to the respiratory chain through the ETF, do not lead to different rates of H_2_O_2_ generation.

In flavoenzymes in which the prosthetic FADH_2_ group reduces NAD^+^, such as α-ketogluratate dehydrogenase, ROS generation is stimulated by the absence of NAD^+^ as the typical electron acceptor [17-20]. Although VLCAD does not reduce NAD^+^, this enzyme produces hydroxyacyl-CoA, which then reduces NAD^+^, and the enzymatic activities are coupled. In order to determine if NAD redox state affects ROS generation under our conditions, we measured the effects of reduced and oxidized NAD on palmitoyl-CoA-induced H_2_O_2_ release from permeabilized mitochondria (Figure 7). We found that NADH concentrations of up to 500 µM do not significantly change rates of H_2_O_2_ release from VLCAD (Figure 7A). On the other hand, increasing NAD^+^ levels significantly prevented H_2_O_2_ release (Figure 7B). The increase in ROS release promoted by the lack of an electron acceptor indicates that electron leakage occurs in this enzyme upstream of NAD^+^ reduction, probably at the level of the semiquinone form of the flavin [43]. 

## Discussion

High-fat diets are frequent in human populations, and, as a result, hepatic steatosis is highly prevalent. Although reasonably benign in itself, non-alcoholic steatosis can progress to more serious liver diseases including fibrosis and cancer. Many different reports have associated the progression of steatosis to mitochondrial dysfunction and ROS overproduction [2,4,44-46]. Despite this, to date no studies have focused on mechanisms through which HFD associated with steatosis promote enhanced ROS release.

Here we show that a short-term (one week) HFD leads to increased microvesicular lipid droplet content in hepatocytes (Figure 1). Interestingly, oxidative imbalance was detected (Figure 2A), although we could not observe significant Kupffer cell infiltration to justify an inflammatory process. Furthermore, HFD does not promote overt changes in mitochondrial respiratory function and oxidative phosphorylation evaluated using succinate (Figure 3) or NADH-linked substrates (not shown). This result is consistent with prior data [34] demonstrating that long-term exposure (1-9 months) to the same diet did not alter mitochondrial respiration or membrane potentials. On the other hand, Mantena [47] observed that a 16 week intervention involving a higher-fat diet can promote partial respiratory inhibition and decreased ATP synthesis, and Vial et al. [41] found an inhibition in maximal respiratory rates of mitochondria from the livers of rats kept on a high saturated fat diet for 8 weeks. Despite the lack of changes in electron transfer and coupling, we find that ROS release from mitochondrial fractions of HFD animals are significantly larger when fatty acids are used as substrates (Figures 1, 5, 7) and that levels of carbonylated proteins are higher (Figure 2), a result consistent with long-term studies [34]. This indicates that in steatosis, ROS overproduction precedes changes in bioenergetics.

We then sought to understand the mechanism through which increased levels of H_2_O_2_ are generated in mitochondrial fractions of HFD livers. The most immediate explanation would be an increase in the presence of contaminant peroxisomes in the mitochondrial fraction [42]. Indeed, peroxisomes participate in lipid oxidation, generate H_2_O_2_ as a reduction product, and would be expected to increase in HFD animals. Despite this, we were able to attribute the enhanced H_2_O_2_ production to mitochondrial activity through a combination of experimental findings (Figure 2): (i) H_2_O_2_ release was equal in cytosolic fractions; (ii) percoll-purified HFD mitochondrial fractions (removed of peroxisomes) still exhibited higher H_2_O_2_ release rates than control samples; (iii) no differences in catalase activities or quantities, a reflection of low and equal levels of peroxisomal contamination, were detected between control and HFD mitochondrial suspensions; (iv) higher H_2_O_2_ release rates were observed using palmitoyl-carnitine, a mitochondrially-specific substrate, and (v) the difference between HFD and control samples was fully reversed by the presence of malonyl-CoA, which inhibits mitochondrial uptake of palmitoyl-CoA.

The mechanism through which palmitoyl-CoA leads to a specific increase in mitochondrial ROS release in the HFD group was then sought. Cocco [48] demonstrated that increases in mitochondrial H_2_O_2_ release could result from respiratory inhibition promoted by fatty acids (arachdonic acid, in their studies). However, we found that the concentrations of palmitoyl-CoA used here did not inhibit respiration supported by classical respiratory substrates (results not shown). This may be because of the low concentrations used and the fact that fatty acids were buffered by 0.1% BSA. Furthermore, our results indicate that respiratory rates supported by palmitoyl-carnitine were higher in HFD (Figure 4), thus eliminating the possibility that the increase in ROS release observed in HFD samples is due to respiratory inhibition. Finally, we find that the rate of H_2_O_2_ produced is enhanced even in mitochondria in which the inner membrane was permeabilized by alamethicin (Figures 6B and 7). This eliminates a role for changes in mitochondrial coupling in the enhanced oxidant release observed in HFD mitochondria.

The specific increase in H_2_O_2_ production found with palmitoyl-CoA associated with a lack of changes in mitochondrial electron transport and coupling suggests the generation of oxidants may occur upstream of the electron transport chain. Indeed, adding NADH, which feeds electrons into the electron transport chain, did not change H_2_O_2_ production. On the other hand, NAD^+^, which can drain electrons from upstream dehydrogenases, decreased H_2_O_2_ release (Figure 7), suggesting an involvement of enzymes upstream of NAD^+^-reducing dehydrogenases outside the electron transport chain.

Seifert and colleagues [49] also found that liver mitochondria generate significant quantities of H_2_O_2_ when respiring on palmitoyl-carnitine. They found that mitochondria generate superoxide radicals in the intermembrane space when fatty acid oxidation is stimulated, which suggests a role for electron transfer chain components in this production. However, under our conditions, H_2_O_2_ release was not influenced by membrane potentials, and therefore could also be ascribed to a ROS source upstream of the electron transport chain. Indeed, mitochondria contain a large number of enzymes that catalyze one-electron transfer reactions (notably flavoenzymes) and are capable of promoting monoelectronic reduction of oxygen, generating O_2_^**.** -^ and secondary ROS [16,19]. In particular, glycerol and α-ketoglutarate dehydrogenase have, in the past, been shown to be significant sources of mitochondrial ROS.

We thus hypothesized that a flavoenzyme involved in fatty acid metabolism (either ETF, ETF-DH, or/and acyl-CoA dehydrogenases) was the source of HFD-induced oxidant release in our model. Not surprisingly, we found that various acyl-CoA dehydrogenase isoforms were more highly expressed in mitochondria from HFD animals, in addition to ETF (Figure 5). When the activity of these enzymes was tested using different chain length CoA derivatives [6,24], only the very long chain activity was found to be enhanced (Figure 6A). Indeed, we were able to pinpoint VLCAD as the most probable source of H_2_O_2_ induced by HFD with the finding that release of ROS was specific to palmitoyl-CoA addition to permeabilized or intact mitochondria (Figure 6B, C). We would like to stress that this finding does not eliminate other flavoenzymes as important sources of mitochondrial ROS, just suggests that VLCAD is the most probable source of higher ROS rates found specifically in HFD mitochondria compared to organelles from control animals. Interestingly, the increases in VLCAD activity (Figure 6A) and H_2_O_2_ release (Figures 2D-F, 6B, 5C, 7) are proportional. Furthermore, NAD^+^ inhibited H_2_O_2_ release rates in both control and HFD mitochondria (Figure 7B), maintaining this proportion, which suggests that the enhanced production of H_2_O_2_ is a direct consequence of the enhanced activity of this ROS-producing enzyme. The effects of added NAD^+^ are probably related to more effective oxidation of the VLCAD product hydroxyacyl-CoA, decreasing the half-life of reduced intermediaries within VLCAD and preventing reactions with oxygen leading to ROS production [43].

This is the first description, to our knowledge, that VLCAD may be a significant source of mitochondrial ROS. Interestingly, although all acyl-CoA dehydrogenases display the same catalytic mechanism [5,6], oxygen reactivity is not necessarily equal. SCAD and MCAD do not show significant oxygen affinity [5]. Crystal structures of VLCAD were determined by McAndrew [50] and indicate the catalytic pocket is significantly larger than the other acyl-CoA dehydrogenases, and may not exclude oxygen as efficiently, which could lead to electron leakage from reduced flavin to oxygen [5,24]. As a precedent, an acyl-CoA dehydrogenase from the bacteria *M. elsdenii* can react with oxygen [51], probably because it possesses a phenylalanine instead of a tryptophan residue at position 166, which may protect the dimethylbenzene side of the flavin ring from oxygen [5]. Further studies are necessary in order to determine the structural characteristics that allow VLCAD to produce ROS under our experimental conditions.

Furthermore, although we believe that the HFD-induced increase in ROS release from VLCAD may be justified solely by the enhanced expression and activity of this enzyme, we cannot exclude other modifications to this enzyme’s function which may lead to enhanced electron leakage. Kabuyama and colleagues [52] demonstrated that serine 586 phosphorylation decreases the activity of VLCAD and is associated with oxidative imbalance in HEK293 cells, suggesting that this posttranslational modification may alter its redox properties. In addition, a recent study [53] demonstrated that protein-protein interactions between MCAD and ETF destabilize the flavosemiquinone in ETF, altering electron leakage to oxygen. It is possible similar mechanisms exist for other acyl-CoA dehydrogenases, including VLCAD.

Overall, while we provide evidence for VLCAD-generated ROS induced by HFD, our evidence does not exclude a ROS-generating role for other mitochondrial flavoenzymes, and the molecular mechanism and conditions in which those enzymes produce reactive oxygen species still remains to be fully understood.

## Conclusions

We demonstrate here that a short-term high fat diet leads to increased mitochondrial production of ROS through a previously undescribed source, VLCAD. In addition to building upon previous data highlighting the importance of mitochondrial sources of oxidants distinct from the electron transport chain [16,19], the finding that ROS generation by VLCAD is regulated by diet suggests it may be central toward the maintenance of cellular redox state under different metabolic conditions.
